# Paleomicrobiology of Humans

**DOI:** 10.3201/eid2403.171908

**Published:** 2018-03

**Authors:** Christopher E. Carr

**Affiliations:** Massachusetts Institute of Technology, Cambridge, Massachusetts, USA; Massachusetts General Hospital, Boston, Massachusetts, USA

**Keywords:** Drancourt M, Raoult D. Paleomicrobiology, infectious diseases, tuberculosis, leprosy, cholera, Bartonelloses, paleopathology, *Helicobacter pylori*, epidemics, archaeology, ancient DNA

We study history in part to avoid repeating it. This fascinating book (Figure) reveals our past and informs our future. Each chapter is like an episode in a televised season of the kind of crime procedural drama that has become wildly popular with the general public, although here each story is both dramatic and factual, and should appeal to both generalists and specialists.

**Figure:**
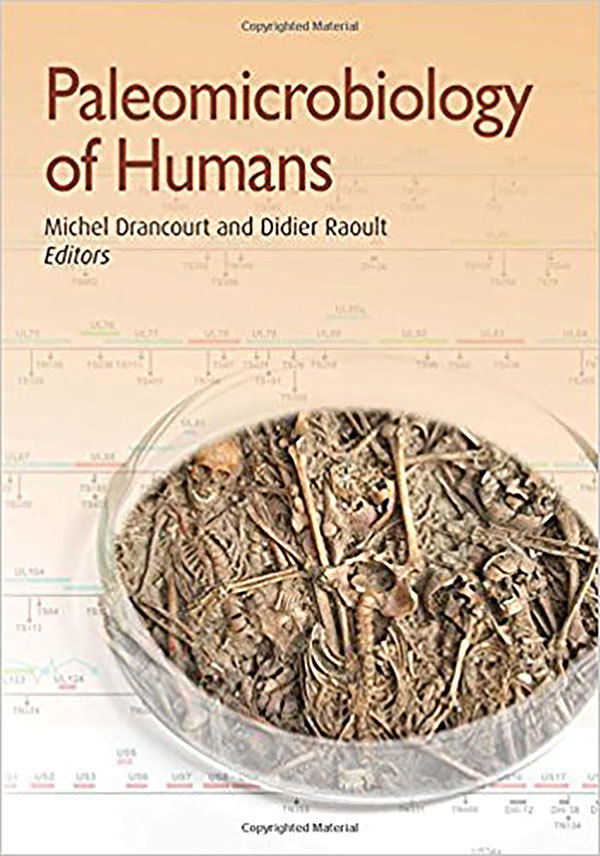
Paleomicrobiology of Humans

Early in this book, we learn how to assess archeological remains, what source materials to target (environmental samples, tissues, bone, teeth, coprolites), and historical and modern ways to investigate them. Common themes emerge: the weakness of negative results (perhaps the “right” site or sample has yet to be found or analyzed); the importance of negative controls and other standard practices; the types of damage experienced by biomolecules over time (*1*); the increasing role of molecular methods; and the continued need for improving them.

Later chapters focus mostly on specific infectious diseases, with some exceptions. High points of the book include evolution of the human immune system and its relationship to archaic humans, ancient antibiotic resistance, and an integrative tale of paleopathology (this one might fit better earlier in the book). The tuberculosis narrative is nicely coupled to the challenges of ancient samples and modern molecular methods. The integrative summary of leprosy raises important questions: Why can’t we culture its causative mycobacterial agents? How will refinement of molecular methods contribute to present-day treatment? One also gets the sense that despite the sample preparation challenges, such methods also have much to contribute to paleoparasitology and present-day eradication efforts. Recent success with antibody-based and nucleic acid (including metagenomic) approaches are mentioned in several chapters, including in the discussion of Malaria; these methods complement skeletal pathophysiology, which can be non-specific.

Then, we move on to discover the roles of trade, slavery, and war in transmission of smallpox. Lingering questions remain: despite great successes, are we at risk for future outbreaks from a pathogen’s relatives? What surveillance is needed? Late in the book, we encounter cholera and the famous John Snow. What will be the impact of climate change, given the linkage between natural disasters and recent outbreaks and epidemics? The final story, about lice, again highlights the importance of molecular methods and their use to infer human history and migrations.

Minor points: In one chapter, figure text is in French, and Raoult's story of lice in plague pandemics is welcomingly personal. The story of Bartonelloses could benefit from more detail on pathogenesis, transmission, and modern risk. And room remains for episodes about influenza and our co-evolutionary history with *Helicobacter pylori*. I also hungered for a final, even if hazy, canvas of the future.

Perhaps next season will go deeper into degradation of biomolecules, nascent molecular methods such as single-molecule or single-cell sequencing and immuno-PCR, and the future of paleomicrobiology. How can we avert the next major infectious disease pandemic? Perhaps with improved global surveillance, as stated by Gardy and Loman (*2*), better informed by paleomicrobiological knowledge. What better place to start than with this book?

## References

[1] Dabney J, Meyer M, Pääbo S. Ancient DNA damage. Cold Spring Harb Perspect Biol. 2013;5:a012567. 10.1101/cshperspect.a012567 23729639 PMC3685887

[2] Gardy JL, Loman NJ. Towards a genomics-informed, real-time, global pathogen surveillance system. Nat Rev Genet. 2018;19:9–20; Epub ahead of print. 10.1038/nrg.2017.88 29129921 PMC7097748

